# Socioeconomic position and urban environments as drivers of antimicrobial resistance? An ecological study in Germany, 2010 to 2019

**DOI:** 10.2807/1560-7917.ES.2025.30.28.2400723

**Published:** 2025-07-17

**Authors:** Regina Singer, Mirco Sandfort, Felix Reichert, Achim Dörre, Jens Hoebel, Anja Klingeberg, Sebastian Haller, Niels Michalski

**Affiliations:** 1 Department of Infectious Disease Epidemiology, Robert Koch Institute, Berlin, Germany; 2 Postgraduate Training for Applied Epidemiology, Robert Koch Institute, Berlin, Germany; 3 ECDC Fellowship Programme, Field Epidemiology path (EPIET), European Centre for Disease Prevention and Control (ECDC), Stockholm, Sweden; 4 Department of Epidemiology and Health Monitoring, Robert Koch Institute, Berlin, Germany; * These authors contributed equally to this work and share last authorship.

**Keywords:** Acinetobacter, Antimicrobial drug resistance, Enterobacterales, Meticillin-resistant *Staphylococcus aureus*, Regional disparities, Socioeconomic factors

## Abstract

**BACKGROUND:**

Germany lacks comprehensive studies on the relationship between socioeconomic position (SEP) and antimicrobial resistance (AMR).

**AIM:**

We assessed the association between area-level SEP and AMR infection and colonisation in Germany.

**METHODS:**

In an ecological study design, we analysed statutory notifications of invasive meticillin-resistant *Staphylococcus aureus* (MRSA, n = 34,440) in 2010−2019, and colonisations and infections with carbapenem-resistant *Acinetobacter* spp. (CRA, n = 1,979) and Enterobacterales (CRE, n = 10,825) in 2017−2019. Area-level SEP was measured by the German index of socioeconomic deprivation (GISD), incorporating education, employment and income data. A multilevel Poisson regression analysis estimated the association between AMR incidence and GISD at district level, adjusting for age, sex, notification year and urbanisation degree.

**RESULTS:**

Median ages of patients with carbapenem-resistant bacteria were between 66 (CRA colonisation) and 69 years (CRE infection). For MRSA infections, the median age was 74 years. Across each pathogen, approximately two thirds of patients were male. Estimated MRSA incidence was almost five times higher in districts with lowest vs highest area-level SEP (incidence rate ratio, IRR: 4.8; 95% CI: 2.8–8.2). This association was strongest in large cities (IRR: 9.1; 95% CI: 2.7–30.9), and sparsely populated rural districts (IRR: 6.5; 95% CI: 2.8–15.0). Associations of CRA (IRR: 0.6; 95% CI: 0.3–1.2) and CRE (IRR: 0.9; 95% CI: 0.6–1.4) infections with SEP were not statistically significant.

**CONCLUSION:**

Lower area-level SEP and degree of urbanisation were associated with MRSA incidence, however, no associations were uncovered between SEP and CRA or CRE infections. Further individual-level research could explore if health behaviours, living/working conditions or healthcare access explain the findings. Socioeconomic conditions should be considered for AMR prevention and control.

**Key public health message**

**What did you want to address in this study and why?**

In Germany, health inequalities due to socioeconomic position (SEP) have been observed for example concerning health behaviour, life expectancy, cancer risk, and some infectious diseases. No comprehensive study has yet examined if SEP is associated with antimicrobial resistance. We wanted to explore whether populations of districts with lower SEP and of cities or rural areas in Germany more likely had infections with antimicrobial-resistant pathogens.

**What have we learnt from this study?**

Our study found that areas with lower SEP, especially among larger cities and among sparsely populated rural districts, were significantly more likely to have higher rates of infections with meticillin-resistant *Staphylococcus aureus* (MRSA). However, no clear link was observed between SEP and other drug-resistant bacteria, such as carbapenem-resistant *Acinetobacter *spp. and Enterobacterales.

**What are the implications of your findings for public health?**

Our findings suggest that socioeconomic disparities play a critical role in the dissemination of antimicrobial-resistant pathogens, with the extent of this role differing according to level of urbanisation. To improve monitoring, prevention and control antimicrobial-resistant pathogen spread, tailored interventions considering both the degree of urbanisation and socioeconomic factors are essential.

## Introduction

Antimicrobial Resistance (AMR), which makes infections hard to treat, is a growing public health threat worldwide [1]. In Germany, AMR is among the top 10 causes of deaths [2], especially due to infections with meticillin-resistant *Staphylococcus aureus* (MRSA) and carbapenem-resistant Enterobacterales (CRE) [2,3]. While the incidence of MRSA has decreased in the last decade, at least partly due to various infection prevention and control measures (e.g. microbiological screening, intensified surveillance, isolation and decolonisation [4-6]), especially CRE incidences have increased since 2020 [7].

Both MRSA and CRE are prone to healthcare-associated transmission, and colonisation often precedes infection [8-10]. Colonisation by MRSA particularly affects the nasal mucosa and community-transmission (associated with close contact with carriers) and livestock-associated transmission are well described [8,11]. By contrast, CRE primarily colonise the gut and spread via faecal contamination of hands or devices. While community-transmission is increasingly implicated in the spread of CRE, such as with *Escherichia coli*, transmission in healthcare settings is considered the main cause of CRE transmission overall [9,10]. In low-incidence countries in Europe, colonisation and infection are often associated with travel to or previous residence in high-incidence countries [12-14]. The risk of pathogen transmission of AMR also varies by sociodemographic and environmental characteristics like age, sex, underlying illnesses, as well as social and environmental factors [15].

In European high-resource settings, studies show a social gradient in the prevalence and burden of AMR, with lower socioeconomic conditions typically linked to a higher risk of acquiring drug-resistant pathogens, like MRSA or CRE [16-19]. Individual socioeconomic position (SEP), including income, education, and employment, affects health, risk for infectious diseases and AMR, either independently or combined with other sociodemographic factors [19-22]. In Germany, several characteristics related to health inequalities due to SEP are observed, such as health behaviour, life expectancy and premature mortality, cancer risk, but also risk of infections, such as HIV infection [21,23-25]. Reasons for health disparities due to SEP are manifold, likely multifactorial and might also vary in the direction of the association between SEP and AMR by pathogen type and its transmission. People in lower SEP groups may face overcrowded living conditions, have limited healthcare access, and higher exposure to antimicrobial-resistant pathogens [19]. In parallel, comorbidities linked with lower SEP might increase exposure to healthcare settings and antibiotic consumption.

No comprehensive study has yet explored the association between SEP and AMR across the entire German population. A better understanding of socioeconomic inequalities is important to understand transmission dynamics, to potentially tailor interventions in particularly affected regions or vulnerable groups, and to derive recommendations to the public and policymakers [15].

In this study, we assessed if residence in areas with lower SEP is associated with higher incidence of infection or colonisation with drug-resistant pathogens in Germany. 

## Methods

### Study design

We estimated the incidence of infection and colonisation based on statutory notifications of drug-resistant pathogens in Germany and analysed the association with area-level SEP in all 401 German districts. The strength of these associations was quantified separately according to the different pathogens. Because population-based individual-level data on AMR in combination with indicators of SEP are not available in a nationally representative sample for Germany, we used an ecological study design to assess the association between area-level SEP and AMR at district level. First, a descriptive analysis determined age- and sex-standardised AMR incidences for the German districts. Second, bivariate correlations of area-level SEP and AMR incidence were explored. Third, a multilevel Poisson regression model was calculated at district level to estimate the association between area-level SEP and AMR incidence [26,27].

### Data sources and case definitions

#### Data on antimicrobial resistance

The dependent variable of the study was the incidence of AMR in Germany. We used yearly data on statutory notified cases of MRSA, carbapenem-resistant *Acinetobacter* spp. (CRA) and CRE, transmitted through national surveillance, which fulfil the case definitions based on the German Infection Protection Act [28,29]. Notified cases are assigned to districts based on their districts of residence. The three pathogen groups provide the most comprehensive data on statutory notifiable AMR pathogens in Germany.

Invasive MRSA infections, detected in blood or cerebrospinal fluid (CSF), have been notifiable in Germany since July 2009. Our analysis comprises data from 2010 to 2019.

Infections and colonisations with CRA and CRE became notifiable in May 2016 in Germany. We examined notification data from 2017 to 2019**.** Whether a notification concerned an infection or a colonisation was inferred from the sample or sampling type resulting in CRE or CRA detection. Infections with CRA and CRE were defined by detection in blood, CSF, bronchoalveolar lavage, urine, tracheal secretion, or wound. Colonisations by CRA or CRE were defined by detection only in screening swabs or stool samples. If the type of sampling or sample was not specified in the notification, reported information on infection or colonisation status was used if available. Cases with missing infection/colonisation information were excluded (CRA: n = 147; CRE: n = 1,034) as shown in Supplementary material 1.

The data included age and sex of the patient (only binary data available as male/female), sample type, method and result of laboratory testing, information of healthcare supply (hospital- or outpatient care), information on clinical outcomes (deceased or not) and the residence of the patient by federal state and district.

To reduce regional distortion of incidence calculations due to outbreaks, all cases from notified healthcare associated infection (HAI) outbreaks were excluded (MRSA: n = 1,093; CRA: n = 167; CRE: n = 421). Furthermore, cases were excluded when information on age, sex and district ID was missing (MRSA: n = 78; CRA: n = 2; CRE: n = 10).

At the end of this process, the total of MRSA infection notifications included in the study was 34,440. The totals of infection and colonisation notifications for respectively CRE and CRA were 1,979 CRA notifications (including 1,194 CRA infections) and 10,825 CRE notifications (including 5,237 CRE infections).

#### German index of socioeconomic deprivation

The exposure of interest was area-level SEP defined by the GISD. The index measures the level of average SEP in German regions using administrative data on education, employment, and income in German districts and municipalities from the Indicators, Maps and Graphics on Spatial and Urban Monitoring (INKAR) database [30]. The GISD data used, refer to the territorial status as of 31.12.2019 and include values for the years 1998–2019 [24]. For our analysis we chose data on the 401 districts and independent cities to ensure comprehensive matching with data on AMR cases. The GISD score ranges from 0 to 1, from lowest (0) to highest deprivation (1), reversely equivalent to high to low SEP. For categorisation we used its five quintiles, from higher (1^st^ quintile) to lower SEP (5^th^ quintile).

#### Additional data sources

A directed acyclic graph (DAG) was drawn to conceptually identify relevant variables for the hypothesis regarding the association, as presented in Supplementary material 2 [31].

Population data for Germany were retrieved from the German Federal Statistical Office as of 2019 [32].

Types of settlement structure and degree of urbanisation were available from the German Federal Office for Building and Regional Planning from 2019 [33]. The 401 German districts were categorised in four groups according to number of inhabitants and population density per square kilometres. The four categories included large cities, urban districts, rural districts, and sparsely populated rural area, as previously described [33].

Data on agriculture with livestock and number of animals were available from the German Federal Statistical Office from 2019 [34]. We calculated density of pigs and poultry per 100,000 inhabitants per district.

To reflect utilisation of healthcare services and the morbidity of the population we used data on inpatient density per district. The density on inpatients by district of residence and year per 100,000 inhabitants was calculated for each district with open-source data from the German Federal Statistical Office from 2019 [35].

### Statistical analysis

The available data allowed us to apply a mixed ecological study design incorporating a multiple-group design of age- and sex-cluster within the districts and fixed effects for each year [27]. The study population was the population of Germany, and the level of analysis were districts in Germany (n = 401).

For the descriptive analysis, age group- and sex-specific AMR data were aggregated to absolute case numbers for each district and incidence rates were age- and sex-standardised. For bivariate analysis, MRSA, CRA and CRE incidences were calculated as yearly mean incidence per 100,000 population per district. Area-level SEP was calculated as median of the GISD per district for the years 2010–2019 for MRSA data analysis and for the years 2017–2019 for CRA and CRE data analysis. For the multivariable analysis we used the area-level SEP for each year per district. The degree of urbanisation of each district was categorised into four categories including large cities, urban district, rural district, and sparsely populated district. Patient density and number of pigs and/or poultry per district were calculated per 100,000 population. Numeric variables, tested for normal distribution by the Shapiro−Wilk test, were described as mean and standard deviation if normally distributed, or as median with interquartile range (IQR) if not.

To explore bivariate correlations of area-level SEP and AMR incidence, Pearson’s correlation coefficient r was calculated, and scatterplots were drawn with a smoothed fitting curve based on Poisson regression.

To estimate the association between area-level SEP and MRSA, CRA and CRE incidence, we calculated incidence rate ratios (IRR) with 95% confidence intervals (95% CI) using multilevel Poisson regressions, comparing districts with highest SEP to those with lowest SEP. Besides the fixed effects for the area-level SEP, age groups, sex, year, and degree of urbanisation, the models included a random intercept at district level and random slope for the area-level SEP to allow for additional heterogeneity between the districts [26,27]. This model allows for multivariable adjustment of the association between area-level SEP and AMR incidence while considering an age and sex standardisation and different population sizes of the districts. The model and variable selection was evaluated by the likelihood ratio test and based on the models’ Akaike information criterion (AIC) values. We adjusted for age group, sex, year of notification and degree of urbanisation. As a sensitivity check we explored the association for varying degrees of urbanisation.

The analyses were performed with R Version 4.2.2 [36].

## Results

### Descriptive analysis of notified cases with antimicrobial-resistant pathogens

We included in the study 34,440 cases with MRSA infection between the years 2010 and 2019 (Table 1). For the years from 2017 to 2019, we included 1,979 CRA cases overall with 60% (1,194/1,979) CRA infections and 10,825 CRE cases with 48% (5,237/10,825) CRE infections. In CRE cases the most common notified pathogens were *Klebsiella pneumoniae* (34%; 3,670/10,825), *E. coli* (19%; 2,003/10,825), and *Enterobacter cloacae* (15%; 1,645/10,825).

**Table 1:** Baseline characteristics of cases with notifiable antimicrobial-resistant pathogens in Germany, 2010–2019 (n = 47,244 notifications)

Characteristic	MRSA		Enterobacterales				Acinetobacter			
	Infection		Infection^a^		Colonisation^b^		Infection^a^		Colonisation^b^	
	Number	%	Number	%	Number	%	Number	%	Number	%
	34,440	100	5,237	100	5,588	100	1,194	100	785	100
**Area-level SEP**										
1^st^ quintile (higher SEP)	4,824	14.0	1,405	26.8	1,723	30.8	326	27.4	282	35.9
2^nd^ quintile	5,729	16.6	997	19.0	1,092	19.5	223	18.7	109	13.9
3^rd^ quintile	6,299	18.3	979	18.7	857	15.3	208	17.4	135	17.2
4^th^ quintile	8,713	25.3	1,100	21.0	1,156	20.7	282	23.6	155	19.7
5^th^ quintile (lower SEP)	8,875	25.8	756	14.4	760	13.6	155	13.0	104	13.2
**Sex**										
Female	12,488	36.3	2,111	40.3	2,003	35.8	402	33.7	268	34.1
Male	21,952	63.7	3,126	59.7	3,585	64.2	792	66.3	517	65.9
**Age**										
Median (IQR)^c^	74 (64–81)		69 (57–78)		67 (53–76)		67 (54–76)		66 (51–75)	
**Age group in years**										
< 1	141	0.4	58	1.1	212	3.8	2	0.2	12	1.5
1** **– < 10	101	0.3	80	1.5	124	2.2	9	0.8	15	1.9
10** **– < 20	107	0.3	70	1.3	78	1.4	19	1.6	18	2.3
20** **– < 30	314	0.9	206	3.9	189	3.4	57	4.8	46	5.9
30** **– < 40	537	1.6	204	3.9	270	4.8	64	5.4	43	5.5
40** **– < 50	1,311	3.8	267	5.1	340	6.1	86	7.2	56	7.1
50** **– < 60	3,580	10.4	665	12.7	764	13.7	185	15.5	103	13.1
60** **– < 70	6,578	19.1	1,111	21.2	1,244	22.3	255	21.4	164	20.9
70** **– < 80	11,829	34.5	1,465	28.0	1,484	26.6	356	29.8	210	26.8
*≥* 80	9,879	28.7	1,111	21.2	883	15.8	161	13.5	118	15.0
**Federal state of residence**										
Baden-Württemberg	1,847	5.4	501	9.6	607	10.9	105	8.8	78	9.9
Bavaria	2,748	8.0	709	13.6	651	11.6	161	13.6	77	9.8
Berlin	2,360	6.9	541	10.3	414	7.4	167	14.1	79	10.1
Brandenburg	1,261	3.7	130	2.5	107	1.9	30	2.5	10	1.3
Bremen	241	0.7	56	1.1	32	0.6	8	0.7	4	0.5
Hamburg	412	1.2	167	3.2	195	3.5	54	4.5	50	6.4
Hesse	2,018	5.9	546	10.4	1,054	18.9	136	11.4	146	18.6
Lower Saxony	4,385	12.7	291	5.6	243	4.3	84	7.0	21	2.7
Mecklenburg-Vorpommern	1,129	3.3	41	0.8	29	0.5	10	0.8	5	0.6
North Rhine-Westphalia	10,428	30.3	1,360	26.0	1,144	20.5	285	23.9	196	25.0
Rhineland-Palatinate	1,154	3.4	279	5.3	272	4.9	35	2.9	30	3.8
Saarland	296	0.9	11	0.2	9	0.2	1	0.1	3	0.4
Saxony	2,328	6.8	205	3.9	286	5.1	47	3.9	34	4.3
Saxony-Anhalt	1,579	4.6	140	2.7	243	4.3	20	1.7	6	0.8
Schleswig-Holstein	1,248	3.6	102	2.0	128	2.3	20	1.7	17	2.2
Thuringia	1,006	2.9	158	3.0	174	3.1	31	2.6	29	3.7
**Year of notification**										
2019	1,809	5.3	2,144	40.9	2,326	41.6	377	31.6	276	35.2
2018	2,425	7.0	1,759	33.6	1,970	35.3	448	37.5	285	36.3
2017	2,828	8.2	1,334	25.5	1,292	23.1	369	30.9	224	28.5
2016	3,196	9.3	NA	NA	NA	NA	NA	NA	NA	NA
2015	3,639	10.6	NA	NA	NA	NA	NA	NA	NA	NA
2014	3,702	10.7	NA	NA	NA	NA	NA	NA	NA	NA
2013	4,344	12.6	NA	NA	NA	NA	NA	NA	NA	NA
2012	4,486	13.0	NA	NA	NA	NA	NA	NA	NA	NA
2011	4,221	12.3	NA	NA	NA	NA	NA	NA	NA	NA
2010	3,790	11.0	NA	NA	NA	NA	NA	NA	NA	NA
**Degree of urbanisation^d^**										
Large city	11,653	33.8	2,192	41.9	2,429	43.5	527	44.1	398	50.7
Urban district	11,564	33.6	1,932	36.9	2,100	37.6	446	37.4	277	35.3
Rural district	5,964	17.3	557	10.6	587	10.5	119	10.0	55	7.0
Sparsely populated rural district	5,259	15.3	556	10.6	472	8.4	102	8.5	55	7.0
**Hospitalisation status**										
Outpatient	2,020	5.9	682	13.0	269	4.8	139	11.6	71	9.0
Inpatient	31,222	90.7	4,244	81.0	5,032	90.1	971	81.3	669	85.2
Missing	1,198	3.5	311	5.9	287	5.1	84	7.0	45	5.7
**Outcome**										
Not deceased	29,973	87.0	4,921	94.0	5,328	95.3	1,110	93.0	746	95.0
Deceased	3,765	10.9	263	5.0	209	3.7	74	6.2	29	3.7
Missing	702	2.0	53	1.0	51	0.9	10	0.8	10	1.3

IQR: interquartile range; MRSA: meticillin-resistant *Staphylococcus aureus*; NA: not applicable/data not available; SEP: socioeconomic position.

^a^ Infection defined as pathogen found in blood, cerebrospinal fluid, bronchoalveolar lavage, urine, tracheal secretion, wound or, when that information was missing, if samples were indicated as infected.

^b^ Colonisation defined as pathogen found in screening swabs or stool samples, or, when that information was missing, if samples were indicated as colonised.

^c^ Numbers in this row do not indicate counts and percentages, but the median year and IQR per pathogen group.

^d ^ Large cities: cities with ≥ 100,000 inhabitants; urban districts: ≥ 50% of the population lives in large or medium-sized towns, and population density is ≥ 150 inhabitants/km²; or population density outside these towns is ≥ 150/km²; rural districts: ≥ 50% of the population lives in large or medium-sized towns but overall density is < 150/km²; or < 50% lives in such towns but population density outside them is ≥ 100/km²; sparsely populated rural districts: < 50% of the population lives in large or medium-sized towns, and population density outside them is < 100/km².

At country level, the number of MRSA infections increased until 2012 and decreased constantly afterwards. The number of CRA cases first increased from 2017 to 2018 and then decreased again in 2019, whereas CRE cases have been constantly increasing since the introduction of the statutory notification (Table 1). Regarding sex, the ratio of male vs female patients was similar across the pathogens, confirming previous study results in which men present proportionally around two thirds of the cases [37] (Table 1). Concerning age, older (≥ 70-year-olds) and youngest (< 1-year-olds) age groups were most affected, especially for MRSA and CRE infections (Table 2). 

**Table 2:** Multivariable analysis of incidence of MRSA, CRA and CRE infections associated with the area-level SEP and other predictors^a^, Germany, 2010–2019 (n = 47,244 patients)

Characteristic	MRSA infections	CRA infections	CRE infections
	IRR (95% CI)	IRR (95% CI)	IRR (95% CI)
Area-level SEP			
GISD score^b^	4.77 (2.79–8.16)	0.63 (0.34–1.18)	0.90 (0.60–1.36)
Year of notification			
2010	Reference	NA	NA
2011	1.12 (1.07–1.17)	NA	NA
2012	1.19 (1.14–1.25)	NA	NA
2013	1.13 (1.08–1.18)	NA	NA
2014	0.96 (0.91–1.00)	NA	NA
2015	0.93 (0.88–0.97)	NA	NA
2016	0.82 (0.78–0.87)	NA	NA
2017	0.71 (0.67–0.75)	Reference	Reference
2018	0.60 (0.57–0.64)	1.22 (1.06–1.40)	1.26 (1.15–1.39)
2019	0.44 (0.42–0.47)	1.01 (0.88–1.17)	1.50 (1.37–1.65)
Sex			
Female	Reference	Reference	Reference
Male	2.30 (2.25–2.35)	2.26 (2.00–2.55)	1.53 (1.42–1.64)
Age group in years			
< 1	14.11 (10.97–18.14)	0.97 (0.23–4.18)	8.16 (5.57–11.96)
1 – < 10	1.14 (0.87–1.49)	0.52 (0.23–1.14)	1.29 (0.90–1.83)
10 – < 20	Reference	Reference	Reference
20 – < 30	2.30 (1.84–2.86)	2.18 (1.30–3.67)	2.04 (1.50–2.76)
30 – < 40	3.91 (3.18–4.81)	2.24 (1.34–3.74)	1.80 (1.33–2.44)
40 – < 50	7.94 (6.52–9.67)	3.41 (2.08–5.59)	2.54 (1.89–3.40)
50 – < 60	20.78 (17.15–25.19)	5.70 (3.56–9.14)	4.12 (3.13–5.42)
60 – < 70	51.49 (42.54–62.33)	10.61 (6.66–16.91)	7.85 (6.00–10.28)
70 – < 80	107.57 (88.92–130.12)	19.95 (12.58–31.64)	12.60 (9.64–16.47)
*≥* 80	173.37 (143.29–209.76)	14.10 (8.77–22.67)	15.23 (11.63–19.96)
Degree of urbanisation			
Large cities	1.35 (1.08–1.70)	2.00 (1.07–1.97)	1.45 (1.17–1.79)
Urban district	0.99 (0.82–1.21)	1.45 (1.43–2.79)	1.20 (1.00–1.46)
Rural district	Reference	Reference	Reference
Sparsely populated rural district	1.00 (0.82–1.21)	1.01 (0.70–1.44)	1.19 (0.97–1.47)

CI: confidence interval; CRA: carbapenem-resistant *Acinetobacter* spp.; CRE: carbapenem-resistant Enterobacterales; GISD: German index of socioeconomic deprivation; IRR: incidence rate ratio; MRSA: meticillin-resistant *Staphylococcus aureus*; NA: not applicable/data not available; SEP: socioeconomic position.

^a^ Adjusted for age group, sex, year of notification, and degree of urbanisation.

^b^ GISD score ranging 0–1, with 0 being the score of the district with the highest socioeconomic position and 1 the score of the district with the lowest socioeconomic position.

About half of the MRSA cases (50%) were found in areas with lower SEP (4^th^ and 5^th^ quintile; Table 1). For CRA and CRE the distribution regarding SEP levels was two-tiered. The highest percentage of the CRA and CRE cases were found in districts with high SEP (1^st^ quintile), but also a high proportion was found in districts with lower SEP (4^th^ quintile). More than two thirds of the AMR cases were notified in large cities and urban districts, which also constitute the highest population number and density. Only a small number of the cases in all categories were notified as outpatient cases.

### Characterisation of the districts

#### Area-level socioeconomic position

**Figure 1:**
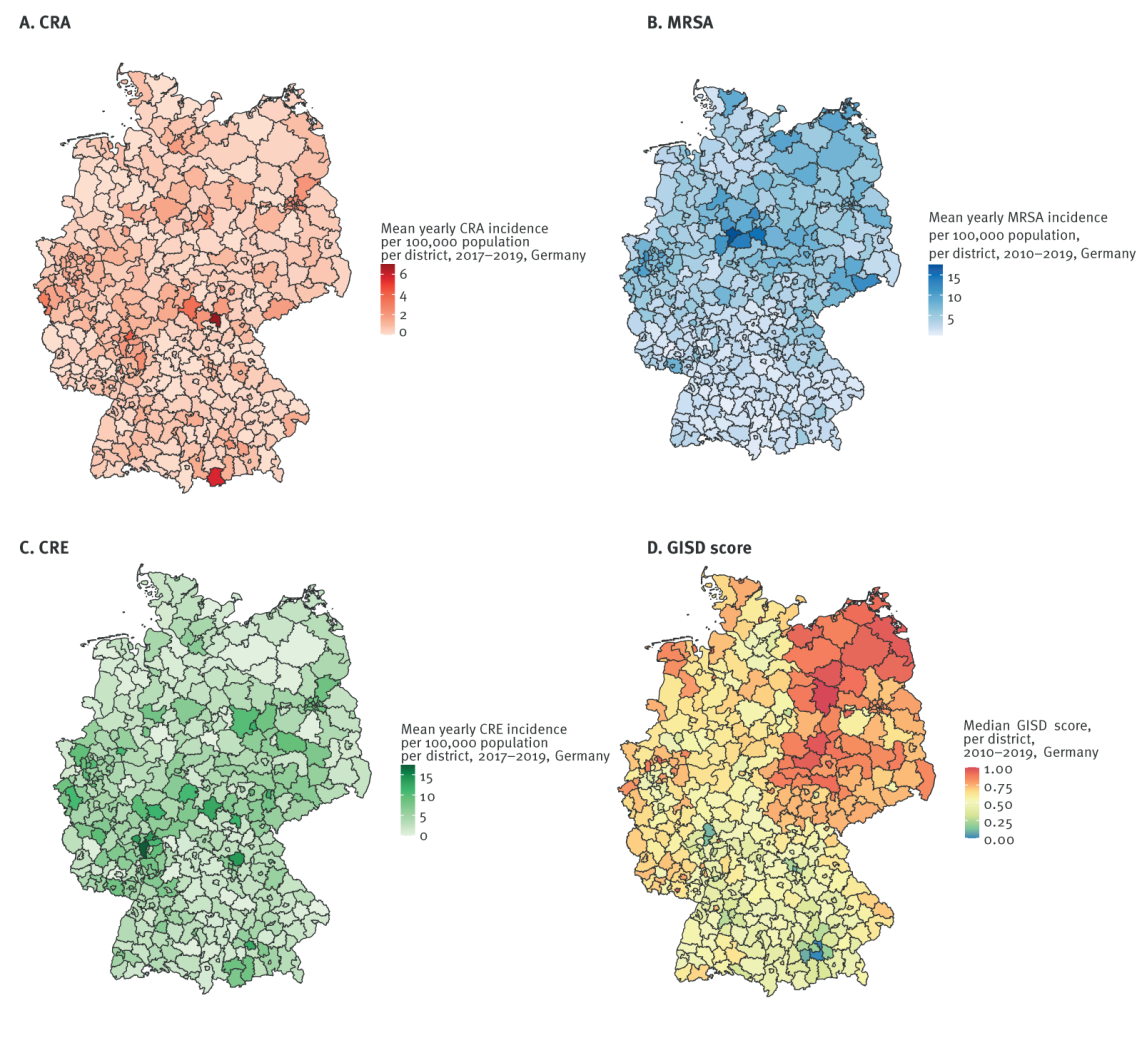
Mean yearly notification incidence per 100,000 population and per district with (A) CRA, (B) MRSA, (C) CRE, and (D) median score of the German index of socioeconomic deprivation (GISD), Germany, 2010–2019 (n = 401 districts)

The SEP measured by GISD showed a north−south and east−west gradient over the districts in Germany (Figure 1).

CRA: carbapenem-resistant *Acinetobacter* spp.; CRE: carbapenem-resistant Enterobacterales; GISD: German index of socioeconomic deprivation; MRSA: meticillin-resistant *Staphylococcus aureus*.

#### Meticillin-resistant *Staphylococcus aureus*

**Figure 2:**
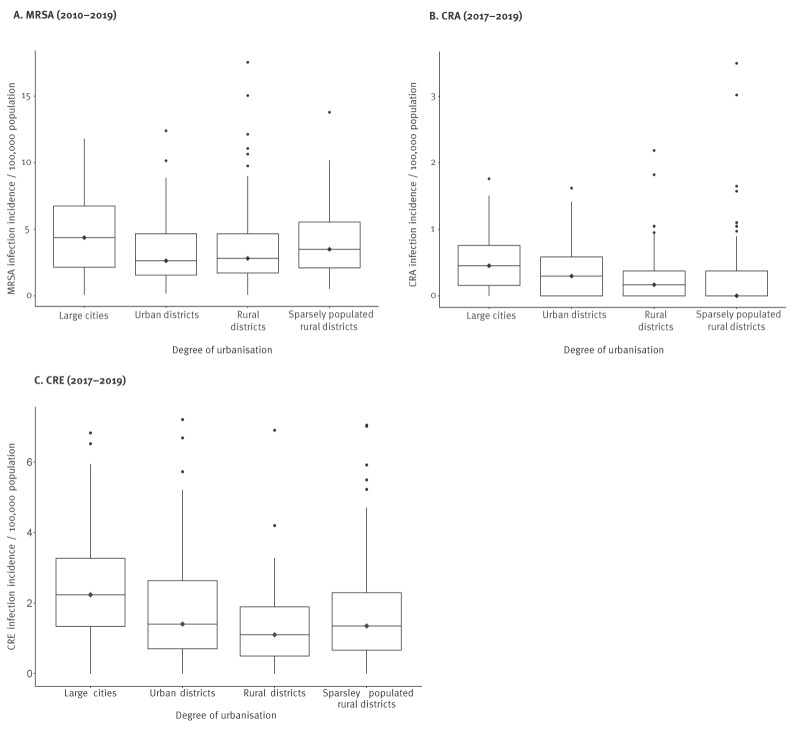
Boxplot with median lines of yearly notification incidences’ means over the study period of (A) MRSA, (B) CRA and (C) CRE infections per 100,000 population, per district, stratified by degree of urbanisation, Germany (n = 401 districts)

The median of the yearly mean incidence of MRSA per 100,000 population per district between the years 2010 and 2019 was 3.1 per 100,000 for the whole country (IQR: 1.8–5.2/100,000), ranging between 0.08 and 17.5 per 100,000 population in the 401 districts. The highest yearly mean incidence was found in central, north-eastern and western districts of Germany (Figure 1). Districts with lowest incidence were found in southern areas of the country, i.e. 7/10 districts with lowest incidence were found in the federal states of Bavaria or Baden-Württemberg. Over the years, MRSA incidence decreased constantly from 2010 to 2019 in 15 of 16 federal states (data not shown). The highest MRSA incidences found appeared to be in large cities followed by sparsely populated areas (Figure 2).

CRA: carbapenem-resistant *Acinetobacter* spp.; CRE: carbapenem-resistant Enterobacterales; MRSA: meticillin-resistant *Staphylococcus aureus*.

#### Carbapenem-resistant *Acinetobacter* species

The yearly mean incidence of all CRA notifications per district varied between 0 and 7.0 per 100,000 population between 2017 and 2019 (median: 0.5/100,000; IQR: 0.2–0.9/100,000), whereas the yearly mean incidence of CRA infections varied between 0 and 3.5 per 100,000 population (median: 0.3/100,000; IQR: 0–0.5/100,000). A total of 85 among 401 districts did not notify any CRA case over the study period. No geographical pattern was visible (Figure 1). Large cities showed the highest mean incidences of CRA infections, the lowest incidences were observed in sparsely populated rural areas (Figure 2).

#### Carbapenem-resistant Enterobacterales

The yearly mean incidence of all CRE notifications per district varied between 0 and 17.4 per 100,000 population between 2017 and 2019 (median: 3.3/100,000; IQR: 1.9–5.6/100,000), whereas the yearly mean incidence of CRE infections varied between 0 and 7.2 per 100,000 population (median: 1.5/100,000; IQR: 0.7–2.4/100,000). Six of 401 districts did not notify any case in the study period. Similar to CRA notifications, there is no apparent geographical pattern (Figure 1). The distribution of the incidence of CRE infections by degree of urbanisation showed a similar pattern to CRA infections, with the highest median incidence in large cities and urban districts (Figure 2).

#### Antimicrobial resistance and area-level socioeconomic position

The bivariate correlation between the yearly mean incidence of MRSA and the median of area-level SEP in the years from 2010 to 2019 showed a moderate positive linear relationship with r = 0.47 (p-value < 0.001). Stratified by degree of urbanisation, the correlation coefficient ranges from r = 0.37 in urban districts to r = 0.59 in large cities. In the multivariable analysis we found that MRSA incidence was significantly higher in districts with lower SEP (Table 3). Contrasting the extremes of the area-level socioeconomic distribution while considering the areas in-between for estimation, MRSA incidence was estimated to be almost five times higher in districts with the lowest than in districts with the highest area-level SEP (IRR: 4.77; 95% CI: 2.79–8.16) (Table 2). In stratified analysis by degree of urbanisation, the highest IRR was observed in large cities, followed by sparsely populated rural areas (Table 3).

**Table 3:** Incidence of MRSA infections associated with area-level SEP, by degree of urbanisation^a^, Germany, 2010–2019

Variable	Large cities	Urban district	Rural district	Sparsely populated rural district
	IRR (95% CI)	IRR (95% CI)	IRR (95% CI)	IRR (95% CI)
GISD score^b^	9.09 (2.67–30.90)	4.11 (1.75–9.64)	1.04 (0.24–4.59)	6.45 (2.79–15.0)

CI: confidence interval; GISD: German index of socioeconomic deprivation; IRR: incidence rate ratio; MRSA: meticillin-resistant *Staphylococcus aureus*; SEP: socioeconomic position.

^a^ Adjusted for age group, sex, year of notification.

^b^ GISD score ranging 0–1, with 0 being the score of the district with the highest socioeconomic position and 1 the score of the district with the lowest socioeconomic position.

The bivariate correlation between the yearly mean incidence of CRA infections and colonisations and area-level SEP from 2017 to 2019 was not significant (r = − 0.04 and r = − 0.03 both with a p-value > 0.05, respectively). Similarly, the correlation for CRE infections and colonisations was also not significant (r = − 0.002 and r = − 0.02 with p-value > 0.05, respectively). Multivariable analysis revealed no statistically significant association between area-level SEP and the incidence of CRA or CRE infections (Table 2) and colonisations, as detailed in Supplementary material 3. The distribution of CRA and CRE infections by age, sex, and degree of urbanisation was similar to MRSA, showing higher IRR in males, the youngest and oldest age groups, and large cities.

Further analysis of potential covariables, including patient density and pig/poultry density, showed moderate correlations with the degree of urbanisation, increasing with rurality (r = 0.4 and r = 0.2, both p-values < 0.001, respectively). Since urbanisation was estimated as a collider for these variables, as indicated by the DAG, they were not included in the final model. Including them in a separate multilevel model, the association between area-level SEP and MRSA incidence remained similar (IRR: 3.55; 95% CI: 2.02–6.25). However, the association for CRA (IRR: 0.44; 95% CI: 0.21–0.94) and CRE (IRR: 0.58; 95% CI: 0.35–0.96) infections became negative, suggesting higher incidence rates in districts with higher area-level SEP.

## Discussion

In our study we found evidence that districts with lower area-level SEP are associated with a higher incidence of MRSA infection. Stratified by degree of urbanisation, this association was strongest in large cities and sparsely populated rural areas. For infections and colonisations with CRA and CRE we did not find a significant association with SEP, which confirms previous study results from Germany on this relationship [37,38]. Our results are in line with findings from other settings. For example, a 2012 study in the United Kingdom found an association between MRSA infection and household deprivation [17], while an analysis of income inequality and drug-resistant pathogens in 15 European countries (2003–2010) revealed a strong correlation between MRSA and income inequality, as well as moderate correlations with carbapenem-resistant *E. coli* or *K. pneumoniae* [39]. A study from Spain describing cases of drug-resistant pathogens between 2014 and 2021, found more cases in areas of high deprivation [40]. These findings suggest that especially the relationship between SEP and MRSA observed in Germany may also hold relevance for other European countries, particularly those with similar socioeconomic structures or health system challenges.

Differences in the association between SEP and drug-resistant pathogens may be due to variations in transmission modes and risk factors. Often carried on the skin or in the nose, MRSA spreads through crowding, skin contact, and shared items [41]. Those risk factors are associated with poverty and other indicators of lower SEP [17,42]. Cross-transmission in healthcare settings plays also a crucial role [43]. Regarding this complexity, we excluded outbreak cases due to nosocomial transmission. The strong association between MRSA infections and SEP in large cities may be attributed to higher population density and crowding. The second highest IRR and still strong association in sparsely populated rural areas is consistent with lower area-level SEP in sparsely populated rural districts (r = 0.3, p-value < 0.001). 

When we adjusted for pig/poultry density in our model, we found a higher MRSA incidence in districts with a higher pig/poultry density (IRR: 1.25; 95% CI: 1.05–1.50) as described in Supplementary material 4, confirming previous hypotheses that pig and poultry farming may be associated with higher incidence of MRSA infections in humans [22]. Nasal colonisation with livestock-associated MRSA is present in 77–86% of occupationally exposed farmers working in MRSA-positive facilities [11]. Farmworkers and their families in Germany tend to have a lower SEP. This could explain the strong link between SEP and MRSA cases in rural areas, as this lower SEP group is at a higher risk of MRSA infection compared with others in the same predominantly low-risk environment [44].

On the other hand, a lower SEP could be linked to lower health literacy, that could result in unhealthier lifestyles and comorbidities, both of which may also be influenced by sex [45]. This can increase the risk of hospitalisation and exposure to nosocomial infections with drug-resistant pathogens, like MRSA [42]. An increasing patient density with increasing rurality of the districts (r = 0.4, p-value < 0.001) and also with decreasing SEP (r = 0.6, p-value < 0.001) might explain this relationship further. Accordingly, when we included patient density and pig/poultry density into our model, we found a still positive but lower association between area-level SEP and MRSA incidence (Supplementary material 4). Between the districts a heterogeneity can be assumed e.g. in varying healthcare access, with fewer providers and specialists in rural areas struggling to meet the growing demand from an ageing population and demographic shifts [46]. With our study we may not fully differentiate if the association with SEP is due to increased community exposure to MRSA or increased exposure to healthcare settings, but we present good evidence to assume a combination of both.

In contrast to MRSA, we found no significant association between the incidence of CRA or CRE and area-level SEP in Germany. This also supports a previous study assessing the association between area-level SEP and *K. pneumoniae* in Germany, which did not find a significant association [38]. This difference may be due to the distinct transmission mode of Enterobacterales bacteria, such as extended-spectrum beta-lactamase (ESBL)-producing *E. coli*, which spread via the faecal-oral route [47,48]. Consequently, crowding, a factor linked to lower SEP, may be less relevant. Our descriptive analysis showed similar levels of CRA/CRE cases in both lower and higher SEP districts (Table 1), however the risk factors for acquisition of the pathogens might differ. One reason for the higher percentage of CRA/CRE case notifications in districts with a higher SEP could be a higher risk of acquisition of drug-resistant pathogens during international travel to high incidence countries e.g. Asian or north African countries [14]. People with higher income are likely to travel more frequently, increasing their risk of especially CRE colonisation and infection.

In our additional multilevel analysis, we found a negative association between districts with a high pig/poultry density and the incidence of CRA (IRR: 0.62; 95% CI: 0.46–0.84) and CRE (IRR: 0.75; 95% CI: 0.63–0.90) infections (Supplementary material 4). Unlike MRSA, direct livestock-associated transmission of CRE/CRA seems less prominent, though indirect routes, such as manure-fertilised vegetables, could contribute [22]. ESBL-producing *E. coli* have been found on vegetables and their rates in vegetarians and in the general population were similar [47]. People with higher SEP, despite better health awareness, face similar risks of exposure to ESBLs from food and environment as those with lower SEP. This could help explain the inverse association between SEP and CRA/CRE incidence.

Although we found a strong association between area-level SEP and MRSA infection, this does not allow for conclusions at the individual level, which require further studies [49]. Future research should differentiate between community-acquired, healthcare-associated, and livestock-associated AMR to better understand modes of transmission.

Several limitations must be acknowledged. The low number of CRA cases and the limited time period for CRA and CRE data may have reduced the ability to identify smaller associations. Furthermore, the low number of non-hospitalised individuals did not allow for separate analyses of the link between SEP and infection/colonisation in community settings vs hospital environments. We could not well distinguish between community- and hospital-associated colonisations and infections. Likely not all outbreak cases have been excluded, as some may not have been detected or been documented.

Unobserved covariables and confounder, e.g. antibiotic prescription and consumption, travel, and migration may also influence the association between SEP and AMR. Those variables were not available at district level for our analysis. Furthermore, excess antibiotic use is a main driver of AMR in high income-countries [50]. Guidelines on strategies to ensure rational use of antibiotics (antibiotic stewardship) in hospitals and about rational prescribing in the outpatient sector, aim to lower the emergence of drug-resistant pathogens in Germany. In addition, variations in AMR screening strategies across federal states and districts may occur. However, the multilevel model specification allows for heterogeneity between the districts which would cover e.g. varying testing compliance. These possible confounders across and within districts or and hospitals could not be adjusted for, but their effect is deemed minimal (e.g. testing processes are based on national recommendations). Some level of heterogeneity in SEP within districts needs to be assumed. A few districts correspond to entire cities with more than a million inhabitants. The metric for area-level SEP chosen here does not differentiate SEP beyond the district level. Delineating the association of SEP and AMR risk with city-specific inter-neighbourhood metrics of SEP for example might be insightful in future works.

We excluded cases notified as part of HAI outbreaks, although it could be assumed that the occurrence or dynamics of outbreaks are also associated with SEP. However, including cases of notified HAI outbreaks (i.e. locally detected outbreaks) could have inflated incidence estimates (partially independently from SEP by design) as case detection is more sensitive during outbreaks (e.g. due to screening of contact persons) compared with routine care (based on risk-adapted admission screening or sampling for clinical diagnosis).

## Conclusion

Our results offer key insights for the public and policymakers to address health inequalities, particularly in the context of increasing urbanisation. Socioeconomic disparities and urban development can influence the spread of drug-resistant pathogens, especially in lower SEP areas. Targeted prevention measures, like risk-adapted screening in high-incidence settings, can help reduce MRSA infections. Additionally, health education and campaigns to raise awareness of risk factors can mitigate inequalities and reduce the spread of AMR. Tailored interventions that consider both urban environments and socioeconomic factors are essential for developing equitable, effective strategies to reduce AMR.

## Data Availability

Aggregated data and study protocol will be shared upon request and after review of a research proposal. All data requests should be directed to the corresponding author.

## Note

The author was a fellow of the German Postgraduate Training in Applied Epidemiology (PAE) associated with the ECDC Fellowship Programme. The views and opinions expressed herein do not state or reflect those of ECDC. ECDC is not responsible for the data and information collation and analysis and cannot be held liable for conclusions or opinions drawn.

## Supplementary Data

418000 SupplementaryMaterial
