# Supplementary material for: Socioeconomic position and urban environments as drivers of antimicrobial resistance? An ecological study in Germany, 2010 to 2019
Source: Euro Surveill. 2025 Jul 17;30(28):2400723. doi: 10.2807/1560-7917.ES.2025.30.28.2400723 (PMC12273257; doi:10.2807/1560-7917.ES.2025.30.28.2400723)
Supplement: SupplementaryMaterial [file 24-00723_SupplementaryMaterial.pdf]

Socioeconomic position and urban environments as drivers of antimicrobial resistance? An ecological study in Germany, 2010-2019

This supplementary material is hosted by *Eurosurveillance* as supporting information alongside the article “Socioeconomic position and urban environments as drivers of antimicrobial resistance? An ecological study in Germany, 2010 to2019”, on behalf of the authors, who remain responsible for the accuracy and appropriateness of the content. The same standards for ethics, copyright, attributions and permissions as for the article apply. Supplements are not edited by *Eurosurveillance* and the journal is not responsible for the maintenance of any links or email addresses provided therein.

Supplementary material 1

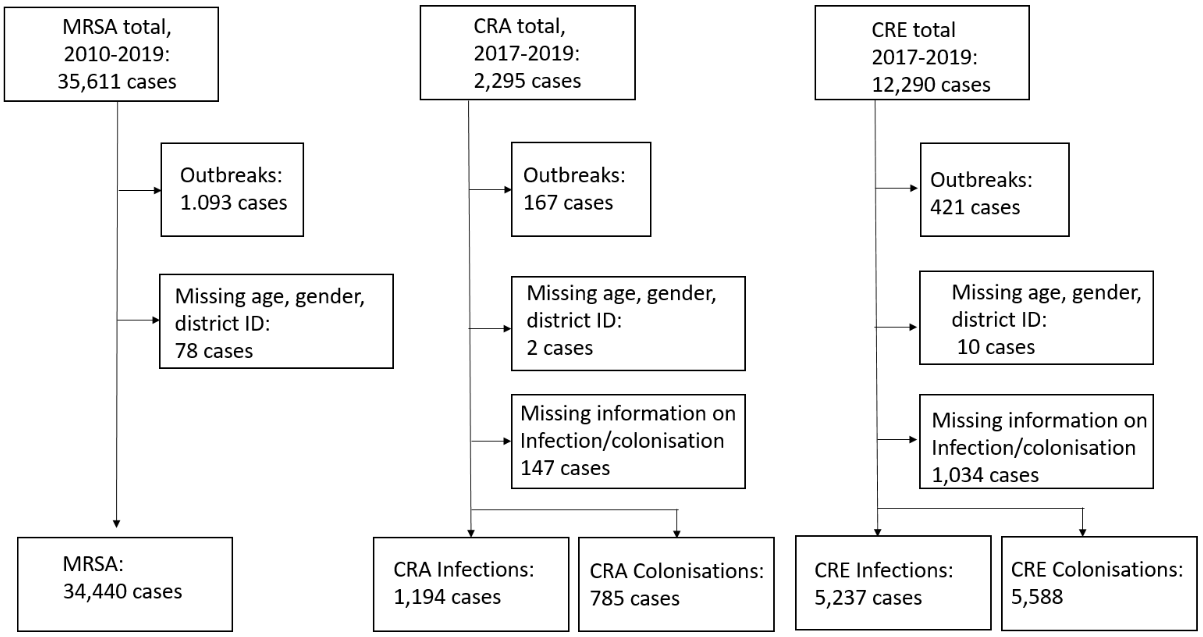

Figure 3: Flowchart of data on AMR case notifications in Germany used for the study: Meticillin-resistant *Staphylococcus aureus* (MRSA), *Acinetobacter spp*. with reduced carbapenem-susceptibility or detection of a carbapenemase determinant (CRA) and Enterobacterales with reduced carbapenem-susceptibility or detection of a carbapenemase determinant (CRE)

Supplementary material 2

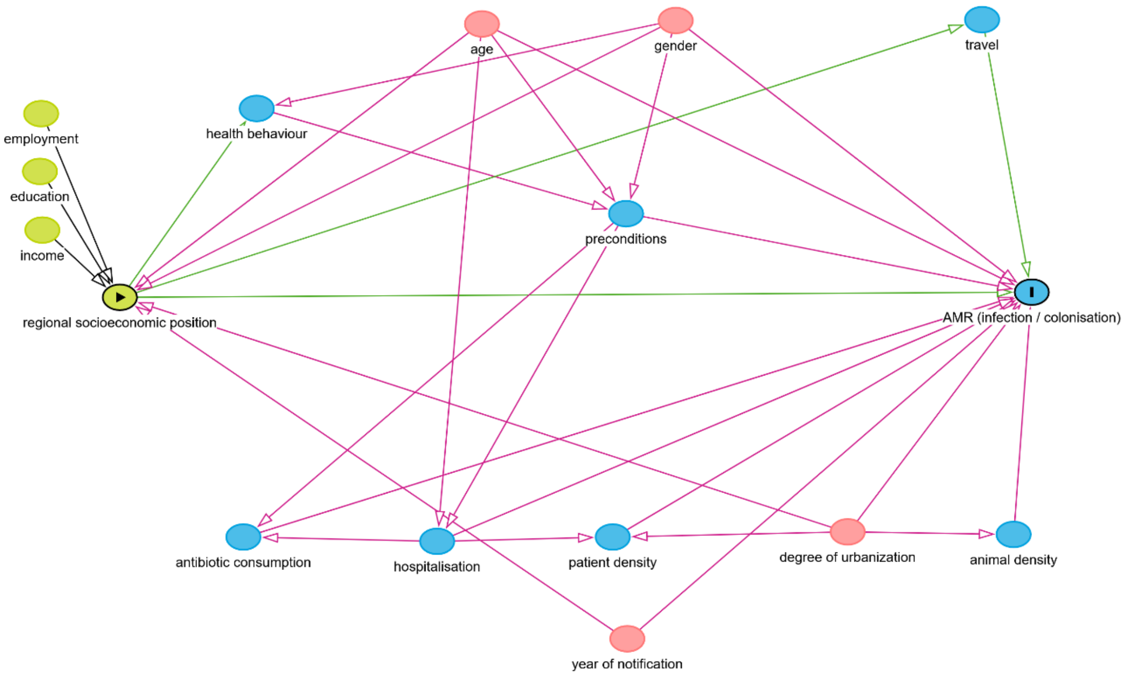

Figure 4: Directed Acyclic Graph of variables

Supplementary material 3

Table 1: Incidence of CRA and CRE colonisations associated with area-level SEP and other predictors*

		CRA colonisations	CRE colonisations
		IRR [CI95%]	IRR [CI95%]
Area-level SEP	GISD score[1]	0.80 [0.23–1.84]	1.24 [0.66–2.35]
Year of notification	2017	Ref.	Ref.
	2018	1.24 [1.04–1.47]	1.51 [1.41–1.62]
	2019	1.21 [1.01–1.44]	1.80 [1.68–1.92]
Sex	Female	Ref.	Ref.
	Male	2.17 [1.87–2.51]	2.05 [1.94–2.17]
Age group in years	<1	6.07 [2.92–12.61]	25.14 [19.39–32.59]
	1 – 9	0.90 [0.45–1.79]	1.73 [1.30–2.29]
	10 – 19	Ref.	Ref.
	20 – 29	1.82 [1.06–3.14]	1.76 [1.36–2.30]
	30 – 39	1.56 [0.90–2.70]	2.30 [1.79–2.96]
	40 – 49	2.21 [1.30–3.76]	3.14 [2.45–4.01]
	50 – 59	3.49 [2.12–5.76]	5.75 [4.56–7.26]
	60 – 69	2.28 [4.48–11.85]	12.54 [9.98–15.77]
	70 – 79	12.67 [7.83–20.50]	20.02 [15.95–25.15]
	≥80	11.05 [6.73–18.13]	17.95 [14.24–22.63]
Degree of Urbanization	Large cities	3.03[2.00–4.59]	2.42 [1.78–3.28]
	Urban district	2.22[1.52–3.23]	1.72 [1.33–2.24]
	Rural district	Ref.	Ref.
	Sparsely populated rural district	1.04 [0.65–1.66]	0.99 [0.74–1.33]

*adjusted for age group, sex, year of notification, and degree of urbanization

[1] GISD score ranging 0–1, with 0 = Score of the district with the highest socioeconomic position to 1= Score of the district with the lowest socioeconomic position

Supplementary material 4

Table 2: Incidence of MRSA, CRA and CRE infections associated with area-level SEP and other predictors*

		MRSA infections	CRA infections	CRE infections
		IRR [CI95%]	IRR [CI95%]	IRR [CI95%]
Area-level SEP	GISD score[1]	3.55 [2.02–6.25]	0.44 [0.21–0.94]	0.58 [0.35–0.96]
Year of notification	2010	Ref.	NA	NA
	2011	1.12 [1.07–1.17]	NA	NA
	2012	1.18 [1.13–1.24]	NA	NA
	2013	1.13 [1.08–1.18]	NA	NA
	2014	0.95 [0.91–1.00]	NA	NA
	2015	0.92 [0.88–0.97]	NA	NA
	2016	0.81 [0.77–0.86]	NA	NA
	2017	0.70 [0.67–0.74]	Ref.	Ref.
	2018	0.60 [0.57–0.63]	1.22 [1.07–1.40]	1.27 [1.15–1.39]
	2019	0.44 [0.41–0.47]	1.01 [0.88–1.17]	1.51 [1.38–1.65]
Sex	Female	Ref.	Ref.	Ref.
	Male	2.30 [2.25–2.35]	2.26 [2.00–2.55]	1.53 [1.42–1.64]
Age group in years	<1	14.11 [10.98–18.15]	0.97 [0.23–4.18]	8.16 [5.56–11.96]
	1 – 9	1.14 [0.87–1.49]	0.52 [0.23–1.14]	1.29 [0.90–1.83]
	10 – 19	Ref.	Ref.	Ref.
	20 – 29	2.30 [1.84–2.86]	2.18 [1.30–3.66]	2.04 [1.50–2.75]
	30 – 39	3.91 [3.18–4.81]	2.24 [1.34–3.74]	1.80 [1.33–2.44]
	40 – 49	7.94 [6.52–9.67]	3.41 [2.08–5.59]	2.54 [1.89–3.40]
	50 – 59	20.78 [17.15–25.19]	5.69 [3.55–9.12]	4.11 [3.13–5.41]
	60 – 69	51.49 [42.54–62.33]	10.58 [6.64–16.86]	7.84 [5.98–10.26]
	70 – 79	107.57 [88.92–130.12]	19.88 [12.53–31.54]	12.57 [9.62–16.43]
	≥80	173.38 [143.30–209.78]	14.04 [8.73–22.58]	15.19 [11.59–19.89]
Degree of Urbanization	Large cities	1.71 [1.231–2.23]	1.73 [1.16–2.58]	1.35 [1.05–1.73]
	Urban district	1.08 [0.89–1.32]	1.30 [0.96–1.77]	1.15 [0.95–1.38]
	Rural district	Ref.	Ref.	Ref.
	Sparsely populated rural district	0.95 [0.79–1.15]	1.02 [0.71–1.45]	1.19 [0.97–1.46]
Patient density	1st Quintile (Lower density)	0.81 [0.66–0.99]	0.93 [0.70–1.22]	0.84 [0.70–1.01]
	2nd – 4th Quintile (Moderate density)	Ref.	Ref.	Ref.
	5th Quintile (Higher density)	1.18 [0.99–1.40]	1.40 [1.04–1.88]	1.29 [1.07–1.54]
Pig and poultry density	1st Quintile (Lower density)	0.90 [0.74–1.11]	1.03 [0.76–1.39]	1.05 [0.86–1.27]
	2nd – 4th Quintile (Moderate density)	Ref.	Ref.	Ref.
	5th Quintile (Higher density)	1.25 [1.05–1.50]	0.62 [0.46–0.84]	0.75 [0.63–0.90]

*adjusted for age group, sex, year of notification, degree of urbanization, patient density, and pig and poultry density

[1] GISD score ranging 0–1, with 0 = Score of the district with the highest socioeconomic position to 1= Score of the district with the lowest socioeconomic position

NA: Not applicable / data not available
